# Development of a fast-crosslinking hydrogel system doped with magnetic mesoporous nanoparticles for sustained fluoride ion release and caries prevention

**DOI:** 10.3389/fbioe.2026.1860798

**Published:** 2026-06-18

**Authors:** Han Lin, Xiaolei Li

**Affiliations:** 1 Guangdong Provincial Key Laboratory of Stomatology, Hospital of Stomatology, Guanghua School of Stomatology, Sun Yat-sen University, Guangzhou, China; 2 Department of Orthodontics, Penn Dental Medicine, University of Pennsylvania, Philadelphia, PA, United States

**Keywords:** anti-caries, controlled fluoride release, drug delievery, hyaluronic acid hydrogels, magnetic mesoporous nanoparticles

## Abstract

**Background:**

Maintaining therapeutic fluoride ion concentrations is essential for preventing enamel demineralization and treating dental defects. However, achieving localized retention and sustained release in the dynamic oral environment remains a significant challenge.

**Aims:**

To develop a multifunctional fluoride ion delivery platform to bridge the gap between high-capacity ion loading and clinical stability at the target site.

**Methods:**

We engineered a hybrid delivery system by integrating fluoride adsorbing magnetic mesoporous nanoparticles (FSMNs) into an ultra-fast photocrosslinkable hyaluronic acid hydrogel matrix (HFMM/PEG). The HFMM polymer, featuring both methacrylic acid and norbornene groups, was synthesized to facilitate rapid *in situ* gelation via thiol ene click chemistry, allowing for sprayable application. The FSMN carriers were constructed with a Fe_3_O_4_ core and a mesoporous silica shell functionalized with chelated Ni^2+^ ions to maximize adsorption. Release kinetics were quantified over 168 h, and *in vitro* biocompatibility was evaluated using human gingival epithelial cells (HGECs).

**Results:**

The HFMM/PEG/FSMN-F^-^ composite significantly decelerated release kinetics, with only 30%–34% of fluoride ions released within 3 h compared to over 60% in traditional fluoride-doped hydrogels. The system maintained a stable and therapeutic fluoride release for up to 168 h (1 week), reaching a cumulative release equilibrium of approximately 90%. Furthermore, *in vitro* evaluations using human gingival epithelial cells (HGECs) confirmed the scaffold’s excellent biocompatibility and cell proliferation properties. *In vitro* anti-*Streptococcus mutans* studies demonstrated that the HFMM/PEG/FSMN-F^-^ had robust caries prevention capacity.

**Conclusion:**

This hybrid hydrogel system bridged the gap between high-capacity ion loading and clinical retention, offering a promising strategy for the long-term prevention of white spot lesions (early caries) in orthodontic and restorative dentistry.

## 1 Introduction

The development of multifunctional biomaterials that combine structural support with the precise delivery of therapeutic ions remains a cornerstone of regenerative medicine (Muzzio et al., 2021). Among these, fluoride ions have gained significant attention due to their pivotal role in enhancing bone mineralization, providing intrinsic antibacterial properties, and preventing dental caries (Gao et al., 2021). However, the therapeutic window for fluoride is narrow; while optimal concentrations promote healing, excessive local levels can lead to cytotoxicity (Gao et al., 2021). Consequently, there is a clinical demand for delivery vehicles capable of extended sustained release while ensuring localized stability at the target site.

We previously developed fluoride ion adsorbing nanoparticles (FSMNs) based on a Fe_3_O_4_@mSiO_2_ core shell structure, functionalized with diethylenetriaminepentaacetic acid (DTPA) chelated Ni^2+^ ions (Li et al., 2021). These FSMNs exhibit a robust fluoride adsorption capacity due to the high affinity coordination between the chelated metal sites and the fluoride ions (F^−^ ions). Despite their superior F^−^ ions loading efficiency, the clinical application of FSMN-F^-^ in dynamic environments, particularly the oral cavity, is severely limited by their low localized retention (Zhao et al., 2020). In the absence of a stabilizing matrix, these nanoparticles were easily cleared by salivary flow or mechanical motion, preventing the maintenance of a therapeutic concentration over the required duration (Khamverdi et al., 2021).

To address this challenge, we proposed encapsulating FSMN-F^-^ within a highly adaptable polymeric scaffold (Li et al., 2024). With the help of a doubly functionalized hyaluronic acid derivative hydrogel grafted with methacrylic acid and norbornene groups (HFMM/PEG), the FSMN-F^-^ can be locked into the target area, overcoming the retention issues associated with free nanoparticles while providing a secondary layer of control over ion diffusion.

In this study, we engineered a hybrid HFMM/PEG/FSMN-F^-^ hydrogel system that merges the high-capacity sequestration of magnetic nanoparticles with the world-leading gelation kinetics of the hyaluronic acid scaffold. This dual network approach created a reservoir plus scaffold system: the FSMN-F^-^ acted as the fluoride reservoir, while the extremely fast-curing hydrogel ensures immediate localized retention and clinical ease of use. We aimed to evaluate the ion release kinetics and assess the efficacy of the HFMM/PEG/FSMN-F^-^ composite for providing sustained fluoride delivery within the complex and dynamic oral environment. In line with this objective, the null hypothesis established for this research was that the HFMM/PEG/FSMN-F^-^ would demonstrate no significant difference in fluoride ion release stability or antimicrobial performance when compared to traditional fluoride delivery systems.

## 2 Materials and methods

### 2.1 Synthesis of FSMNs (Fe_3_O_4_@mSiO_2_-SiCDs@DTPA-Ni^2+^)

The preparation of the FSMN system follows our previously established protocol with specific modifications for ion sequestration (Li et al., 2021). Initially, magnetic Fe_3_O_4_ cores were synthesized by dissolving 0.325 g of anhydrous iron (III) chloride (FeCl_3_) and 0.45 g of sodium citrate dihydrate in 40 mL of ethylene glycol. Upon the formation of an orange-yellow solution, 3.0 g of anhydrous sodium acetate was added to achieve a homogeneous yellow-brown mixture. This solution was heated to 200 °C for 10 h in a 50 mL Teflon-lined hydrothermal reactor. The resulting magnetic particles were washed five times with deionized water and vacuum dried.

Subsequently, 0.1 g of these Fe_3_O_4_ particles was combined with 2.5 mL of deionized water and 10 mL of anhydrous ethanol, and the mixture was ultrasonically dispersed. To this mixture, 2.5 mL of diluted silica-doped carbon dots (SiCDs) solution, 0.1 g of cetyltrimethylammonium bromide (CTAB), 6 mL of deionized water, and 15 mL of anhydrous ethanol were added. Gelation of the silica shell was initiated by injecting 0.4 mL of ammonia solution and 2.5 mL of tetraethyl orthosilicate (TEOS), followed by stirring for 2 h. After magnetic collection and washing, the CTAB template was removed by refluxing in an anhydrous ethanol solution containing ammonium nitrate (0.6 wt%) at 75 °C for 12 h to yield Fe_3_O_4_@mSiO_2_-SiCDs nanoparticles.

For surface functionalization, 0.35 g of these nanoparticles reacted with 0.7 mL of (3-aminopropyl)triethoxysilane (APTES) in 30 mL of isopropanol for 24 h. The aminated product was then dispersed in 36 mL of a 50% v/v acetic acid ethanol solution and refluxed at 80 °C for 16 h with DTPA anhydride. Finally, the Fe_3_O_4_@mSiO_2_-SiCDs@DTPA particles were recovered and stirred with a slight excess of nickel (II) nitrate (Ni(NO_3_)_2_) for 24 h at pH 6.0. The finished FSMNs were collected by freeze-drying.

### 2.2 Preparation of fluoride pre-adsorbed FSMN (FSMN-F^-^)

To prepare the fluoride-loaded carriers, 10 mg of FSMN powder was dispersed in 3 mL of deionized water. Then, 30 mL of a 10 mg/L sodium fluoride (NaF) aqueous solution was added, and the mixture was shaken for 1 h. The particles were collected, washed, centrifuged, and freeze-dried to obtain the final FSMN-F^-^ nanoparticles (Li et al., 2021).

### 2.3 Preparation of HFMM/PEG/FSMN-F^-^ composite hydrogel

The hydrogel matrix preparation was modified based on the ultra-fast crosslinking method reported in our previous work (Li et al., 2024). The hyaluronic acid (HA) molecular weight was MW = 1,000,000 Da, instead of the 100,0000. The synthesis of the doubly functionalized hyaluronic acid derivative (HFMM) was conducted through a sequential three-step modification process. Initially, 5 g of HA was dissolved in 500 mL of deionized water and reacted with 15 mL of methacrylic anhydride added dropwise over 3 h while the system pH was maintained at 8.5 using 5 M sodium hydroxide. Following overnight stirring at 37 °C, the solution was purified via dialysis for 3 days using a 3,000 Da molecular weight cutoff membrane and freeze dried to obtain the methacrylamide modified HA intermediate termed HM. Subsequently, 4 g of HM was dissolved in 1.2 L of 100 mM 2-(N-morpholino) ethanesulfonic acid (MES) buffer at pH 4.5, and the carboxyl groups were activated by adding 11.2 g of 4-(4,6-dimethoxy-1,3,5-triazin-2-yl)-4-methylmorpholinium chloride (DMTMM) for 1 h. Then, 1.5 mL of furan was added dropwise over 5 min and stirred for 24 h at room temperature before the mixture was dialyzed and freeze-dried to produce the furan-modified intermediate hyaluronic acid (HFM). In the final stage, 3 g of HFM was dissolved in 1.2 L of deionized water and reacted with 3 g of maleimide monomer for 24 h at room temperature to construct the norbornene-like groups via a Diels–Alder reaction. The final product was purified through 3 days of dialysis and freeze-dried to yield the white flocculent HFMM solid ready for hydrogel preparation (Li et al., 2024).

The precursor solution was formed by dissolving 0.4 g of HFMM solid and 0.1 g of lithium phenyl-2,4,6-trimethylbenzoylphosphinate (LAP) photoinitiator in 20 mL of deionized water (Li et al., 2024). Once uniform, 0.01 g of FSMN-F^-^ particles was incorporated, followed by the addition of four-arm thiol-terminated polyethylene glycol (PEG) to reach a concentration of 1,000 mg/mL.

For the fluoride ion control group (HFMM/PEG/F^−^), 0.473 mg of NaF powder was added to 20 mL of the HFMM precursor solution. This amount was calculated based on the established adsorption capacity of FSMN-F^-^ to ensure the total fluoride content was equivalent between the experimental and control groups (Li et al., 2021).

### 2.4 Characterization of nanoparticles and hydrogel scaffolds

#### 2.4.1 Scanning electron microscopy (SEM)

The surface morphology of the synthesized FSMN nanoparticles and the internal microarchitecture of the HFMM/PEG/FSMN-F^-^ hydrogels were evaluated using scanning electron microscopy. For nanoparticle imaging, the powder was dispersed in ethanol and deposited onto a silicon wafer. For hydrogel analysis, samples were first freeze dried to preserve their porous structure and then fractured to expose the internal cross section. All samples were mounted on aluminum stubs using conductive carbon tape and sputter coated with a thin layer of gold to minimize charging during imaging. The acceleration voltage was maintained 10 kV to capture high-resolution images of the material porosity and nanoparticle distribution (Li et al., 2021; Li et al., 2024).

#### 2.4.2 Transmission electron microscopy (TEM)

The core-shell architecture of the magnetic mesoporous nanoparticles was confirmed using transmission electron microscopy. The FSMN samples were suspended in deionized water and subjected to ultrasonic treatment for 10 min to ensure uniform dispersion. A single drop of the suspension was placed onto a carbon-coated copper grid and allowed to dry completely at room temperature before examination. Imaging was performed to verify the encapsulated Fe_3_O_4_ core and to measure the thickness and uniformity of the mesoporous silica shell layer (Li et al., 2018).

#### 2.4.3 Nitrogen adsorption desorption analysis

The textural properties of the FSMN carriers, including specific surface area and pore characteristics, were determined via nitrogen adsorption and desorption isotherms at 77 K using a surface area analyzer. Prior to measurement, approximately 100 mg of the sample was degassed at 120 °C under vacuum for at least 6 h to remove any pre adsorbed gaseous species. The specific surface area was calculated according to the Brunauer-Emmett-Teller (BET) method. The total pore volume was estimated based on the amount of nitrogen adsorbed at a relative pressure of 0.99, while the pore size distribution was derived from the desorption branch of the isotherms using the Barrett-Joyner-Halenda (BJH) model (Li et al., 2018).

### 2.5 Determination of fluoride ion release kinetics

The quantification of fluoride release was performed using a colorimetric method based on the national standard HJ 488 2009 (Li et al., 2021). A mixed chromogenic reagent was prepared using fluoride reagent solution, sodium acetate buffer, acetone, and lanthanum nitrate solution in a 3:1:3:3 volume ratio.

A standard curve was generated using sodium fluoride solutions ranging from 0.125 to 3.6 μg in 1 mL of deionized water mixed with 1 mL of the chromogenic reagent. Absorbance was measured at 630 nm after a 30 min incubation.

For the release study, 500 μL volumes of the HFMM/PEG/FSMN-F^-^ and HFMM/PEG/F precursor solutions were placed in centrifuge tubes and irradiated with UV light (395 nm, 50 mW/cm^2^) for 10 s to induce gelation (n = 3) (Li et al., 2021). Each tube received 10 mL of deionized water and was placed on a shaker at 100 rpm. At specific time points, 1 mL of leachate was withdrawn for analysis and replaced with fresh deionized water.

An additional control group utilized the HFMM/PEG/FSMN-F^-^ precursor without UV irradiation to observe the release behavior of the uncrosslinked system under magnetic stirring. All experiments were performed in triplicate and the final release curves were plotted based on the absorbance at 630 nm.

### 2.6 *In vitro* biocompatibility and cytotoxicity assays

#### 2.6.1 Cell culture and maintenance

Human gingival epithelial cells (HGECs) were utilized to evaluate the cytocompatibility of the hydrogel system. The cells were cultured in specialized epithelial cell medium supplemented with 10% fetal bovine serum and 1% penicillin streptomycin. The cultures were maintained in a humidified incubator at 37 °C with a 5% CO_2_ atmosphere. The culture medium was refreshed every 2 days, and cells were passaged upon reaching approximately 80% confluence (Li et al., 2021).

#### 2.6.2 Cytotoxicity of FSMN-F^-^ nanoparticles

To assess the safety of the fluoride-loaded magnetic nanoparticles, HGECs were seeded in 96-well plates at a density of 10^4^ cells per well (n = 3). After 24 h of attachment, the medium was replaced with fresh media containing varying concentrations of FSMN-F^-^ nanoparticles ranging from 10 to 500 μg/mL. After incubation for 24 and 48 h, cell viability was quantified using the Cell Counting Kit-8 (CCK-8) assay (Li et al., 2021). The absorbance was measured at 450 nm using a microplate reader, and viability was expressed as a percentage relative to the untreated control group.

#### 2.6.3 Biocompatibility of HFMM/PEG/FSMN-F^-^ hydrogels

The biocompatibility of the composite hydrogel was evaluated using both extract-based assays and direct contact assays. For the extract assay, sterile hydrogel samples were incubated in culture medium for 24 h at 37 °C to obtain the leachate (n = 3). HGECs were then cultured with this leachate for 48 h (Li et al., 2018).

### 2.7 Evaluation of antibacterial and antibiofilm activity

#### 2.7.1 Bacterial strain and culture conditions

*Streptococcus mutans* UA159 (*S. mutans*) was utilized as the model cariogenic pathogen for all antimicrobial assays. The bacteria were cultured in Tryptone Yeast Extract broth supplemented with 1% glucose at 37 °C in a 5% CO_2_ atmosphere. For biofilm studies, the medium was further supplemented with 1% sucrose to promote the synthesis of extracellular polysaccharides (EPS). The bacterial concentration was adjusted to an optical density of 0.1 at 600 nm, corresponding to approximately 1 × 10^8^ colony-forming units per milliliter (CFU/mL), prior to each experiment (Yue et al., 2021).

#### 2.7.2 Preparation of saliva-coated hydroxyapatite disks

To simulate the tooth enamel surface, hydroxyapatite disks were coated with filtered human saliva to form an experimental pellicle (n = 6). Sterile hydroxyapatite disks were incubated in clarified human saliva for 1 h at 37 °C under constant agitation. Following incubation, the disks were washed with sterile saline to remove unbound proteins. The HFMM/PEG/FSMN-F^-^ precursor solution was then applied to the surface of the saliva-coated HA (sHA) disks using a specialized spray device and immediately stabilized via UV irradiation (395 nm, 50 mW/cm^2^) for 10 s (Liu et al., 2018).

#### 2.7.3 Biofilm inhibition and colony-forming unit (CFU) quantification

The hydrogel-coated sHA disks were placed vertically in 24-well plates containing the *S. mutans* inoculum and incubated for 24, 48, and 72 h (n = 6). Culture medium in the 24-well plates was changed every 12 h. At each time point, the disks were removed and gently washed to eliminate planktonic bacteria. The adhering biofilm and the integrated hydrogel were recovered by transferring the disks into tubes containing sterile saline, sonication for 30 s, and vigorous vortexing. The resulting bacterial suspensions were serially diluted and plated on agar. After 48 h of incubation, the colonies were counted to determine the total CFU per disk (Liu et al., 2018).

#### 2.7.4 Monitoring of biofilm acidogenesis

Since the production of organic acids is a primary virulence factor of *S. mutans*, the culture medium pH was monitored to assess biofilm metabolic activity (n = 6). Culture medium in the 24-well plates was changed every 12 h. At 48 h during the biofilm growth period, 1 mL of the supernatant was withdrawn from each well. The pH was measured with a calibrated micro pH electrode (Yue et al., 2021; Liu et al., 2018).

#### 2.7.5 Confocal laser scanning microscopy (CLSM) analysis

The structural integrity and spatial distribution of the biofilm were visualized using CLSM. *Streptococcus mutans* biofilms were grown in 1% sucrose Tryptone Yeast Extract broth. After 48 h, the live bacteria within the biofilm were stained with SYTO 9 (green fluorescence). The samples were imaged using a confocal laser-scanning microscope with a ×20 water-immersion objective. Three-dimensional reconstructions were generated, and quantitative parameters, including total biomass, biofilm thickness, and the EPS-to-bacteria ratio, were calculated (Yue et al., 2021).

### 2.8 Statistical analysis

All quantitative data from the fluoride release and biocompatibility experiments were expressed as the mean plus or minus the standard deviation. Statistical significance was determined using t-test, one-way analysis of variance (ANOVA) followed by Tukey’s *post hoc* test, and two-way ANOVA followed by Tukey’s/Šídák’s multiple comparisons test. A p value of less than 0.05 was considered statistically significant.

## 3 Results

### 3.1 Morphology and characterization of FSMN nanoparticles and HFMM/PEG/FSMN-F^-^ hydrogel

**FIGURE 1:**
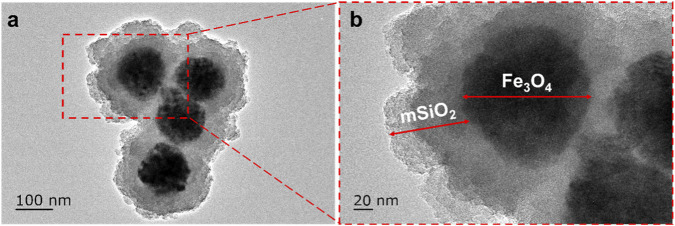
TEM images of FSMN nanoparticles. **(a)** TEM image of FSMN nanoparticles at low magnification. **(b)** TEM image of FSMN nanoparticles at high magnification.

**FIGURE 2:**
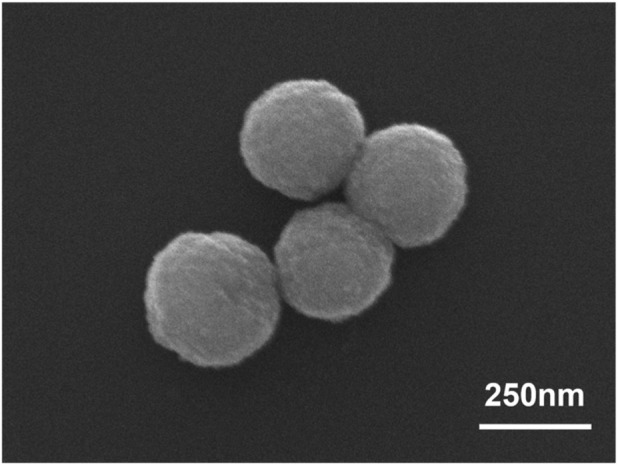
SEM image of FSMN nanoparticles.

As shown in the TEM images (Figure 1), the FSMN nanoparticles possess a well-defined spherical geometry with a distinct core shell architecture and a characteristic rough surface. At high magnification (Figure 1b), the hierarchical structure was clearly visible, consisting of a central magnetic Fe_3_O_4_ core with a diameter of approximately 100 nm and an outer mesoporous silica shell with a thickness of about 75 nm. The total average diameter of the composite nanoparticles was approximately 250 nm. The SEM images (Figure 2) further confirm that the FSMN nanoparticles were uniform and exhibit excellent dispersibility.

This high degree of dispersion suggests that the nanoparticles can remain stable in aqueous media as individual units, which was a critical requirement for a carrier intended for localized drug delivery systems.

**FIGURE 3:**
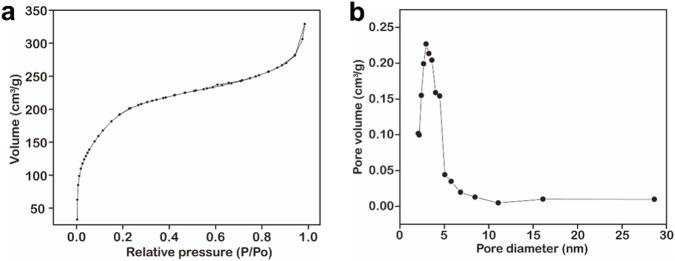
Nitrogen adsorption/desorption isotherm **(a)** and pore-size distribution curve **(b)** of FSMN nanoparticles.

The textural properties of the FSMN nanoparticles were quantified through nitrogen adsorption and desorption measurements. The resulting isotherm (Figure 3a) exhibits type IV classification with a clear hysteresis loop, a hallmark of a mesoporous surface. Using BET and BJH analytical methods, the specific surface area was calculated to be 282.34 m^2^/g, and the total pore volume was 0.63 cm^3^/g. Furthermore, the pore size distribution curve (Figure 3b) indicates a narrow distribution with an average mesopore diameter of 3.1 nm. These values represent a significant increase in surface area compared to pristine iron oxide nanoparticles, providing a high density of functional sites for subsequent Ni^2+^ chelation and fluoride ion adsorption.

**FIGURE 4:**
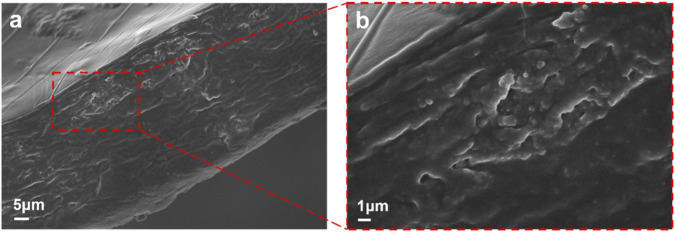
SEM images of HFMM/PEG/FSMN-F- hydrogel. **(a)** Low magnification images demonstrating the immobilization and distribution of the FSMN-F- within the porous hydrogel matrix base. **(b)** High magnification images revealing the detailed surface morphology and interfacial integration within the polymer network.

The integration of these nanoparticles into the hydrogel matrix was observed via SEM (Figure 4). The cross-sectional images of the HFMM/PEG/FSMN-F^-^ hydrogel reveal that the FSMN-F^-^ nanoparticles were successfully immobilized and uniformly distributed within the porous network of the HFMM/PEG scaffold. This successful encapsulation ensures that the nanoparticles were retained at the target site by the ultra-fast curing hydrogel matrix.

### 3.2 HFMM/PEG/FSMN-F^-^ fluoride ion release kinetics

The fluoride ion adsorption capacity of the FSMN system was previously established at 21.4 mg/g based on the specific coordination between the ions and the chelated metal sites. To quantify the release behavior from the composite hydrogel, a colorimetric standard curve was established. The absorbance at 630 nm showed a strong linear correlation with the fluoride ion concentration (C) according to the equation over a range of 0.0625–1.8 μg/mL.

$$A=0.3372\times C+0.0735\;\left(R^{2}=0.9987\right)$$

**FIGURE 5:**
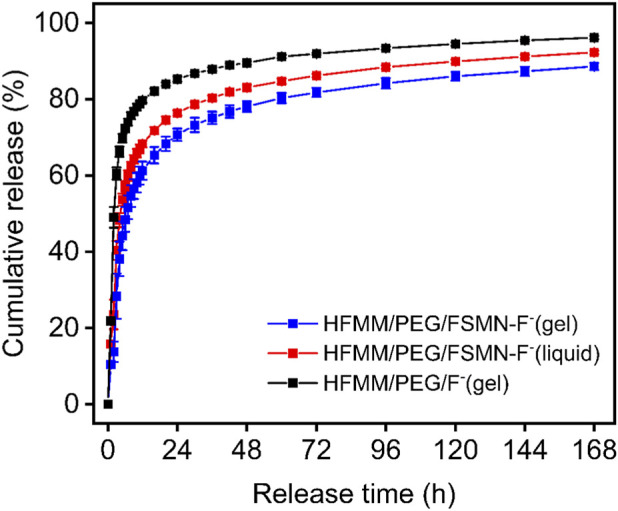
F^−^ ions release curve of HFMM/PEG/FSMN-F^-^ hydrogel, HFMM/PEG/FSMN-F^-^ liquid and HFMM/PEG/F^−^ hydrogel. At each time point after 0 h, significant differences in the cumulative release percentage were observed among the groups (n = 3, *P* < 0.05, two-way ANOVA with Tukey’s multiple comparisons test).

The cumulative release profiles for the HFMM/PEG/FSMN-F^-^ hydrogel, free FSMN-F^-^ nanoparticles, and the HFMM/PEG/F^−^ control group were presented in Figure 5. While all three groups exhibited sustained release, the release kinetics differed significantly depending on the delivery vehicle. The HFMM/PEG/F^−^ hydrogel, which contained free sodium fluoride within the matrix, exhibited the most rapid release. This group experienced a pronounced burst release within the first 3 h, with approximately 40%–43% of the total fluoride ions escaping into the medium. It took 8 h to reach a 60% cumulative release. The fact that the cumulative release did not reach 100% suggests that the Ni^2+^ chelation sites on the FSMN surface exerted strong adsorption forces, preventing some ions from being released by simple passive diffusion.

The most superior sustained release performance was observed in the HFMM/PEG/FSMN-F^-^ hydrogel system. This composite material only released 30%–34% of its fluoride load within the first 3 h and required approximately 12 h to achieve a 60% cumulative release. This significant deceleration of release kinetics was attributed to the dual barrier effect: the fluoride ions must first overcome the chemical affinity of the FSMN nanoparticles and then diffused through the crosslinked HFMM/PEG hydrogel network. The cumulative release eventually approached an equilibrium at approximately 90%. This reservoir plus scaffold strategy effectively eliminated the initial burst release and provided a more stable, long-term therapeutic window, which was essential for applications in dynamic environments like the oral cavity.

### 3.3 Ultra-fast gelation and comparison with traditional hydrogels

**FIGURE 6:**
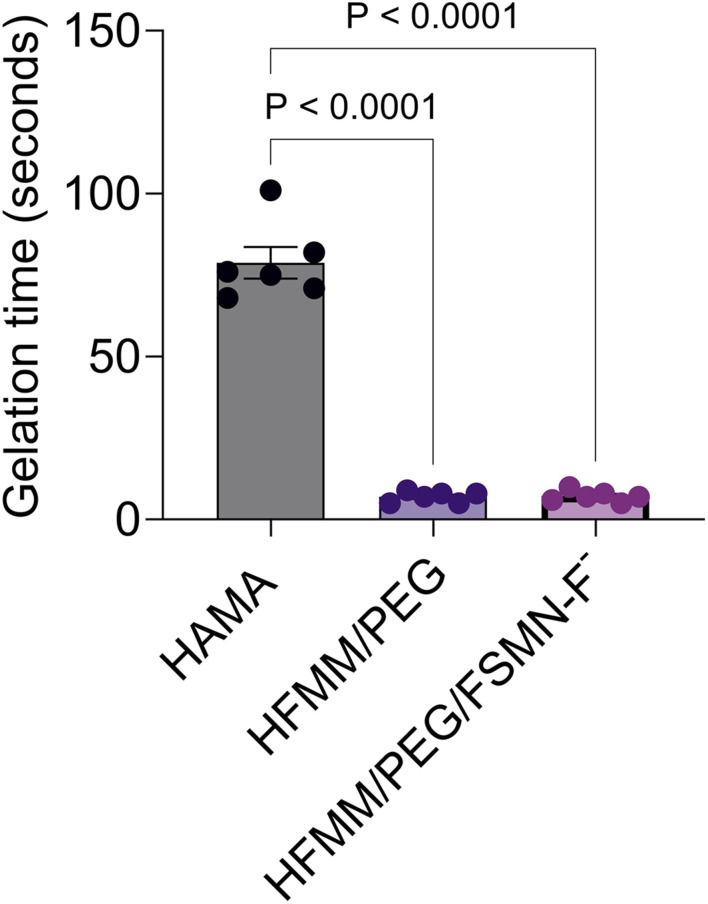
Gelation time of HAMA hydrogel, HFMM/PEG under UV irradiation. (n = 6, one way ANOVA with Tukey’s *post hoc* comparisons).

Under standard UV irradiation (395 nm, 50 mW/cm^2^), the HFMM/PEG precursor solution underwent a phase transition from liquid to solid hydrogel within less than 10 s (Figure 6). In contrast, traditional HAMA hydrogels often require more than 60 s of exposure to achieve comparable mechanical stability. This speed was attributed to the high reactivity of the norbornene groups toward the thiol radicals and the complementary crosslinking provided by the methacrylic groups.

### 3.4 In vitro biocompatibility and cytotoxicity on HGECs

#### 3.4.1 Cytotoxicity of FSMN-F^-^ nanoparticles

**FIGURE 7:**
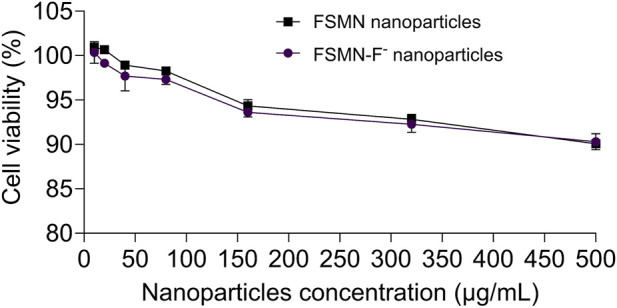
Biocompatibility of FSMN-F^-^ nanoparticles. From 10 to 500 μg/mL, there is no significant differences in the cell viability between FSMN and FSMN-F^-^ groups (n = 3, *P* > 0.05, two-way ANOVA with Šídák’s multiple comparisons test).

The CCK eight assay results indicated that the FSMN-F^-^ nanoparticles possessed excellent cytocompatibility (Figure 7). When HGECs were exposed to nanoparticle concentrations ranging from 10 to 500 μg/mL, the cell viability remained above 90% after 48 h of incubation. These findings demonstrated that the nickel ions chelated within the DTPA functionalized mesoporous silica were stably sequestered and do not leach in quantities sufficient to cause heavy metal toxicity. Additionally, the fluoride ions pre-adsorbed onto the FSMNs were released at a controlled rate that stays within the therapeutic window, avoiding the cytotoxic effects associated with high-concentration fluoride bursts (Islam et al., 2025).

#### 3.4.2 Cell proliferation in the HFMM/PEG/FSMN-F^-^ composite hydrogel leachate

**FIGURE 8:**
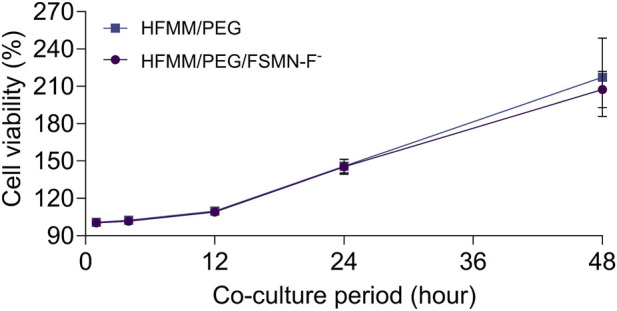
Biocompatibility of HFMM/PEG/FSMN-F^-^ composite hydrogel leachate. From 1 to 48 h, there is no significant differences in the cell viability between HFMM/PEG and HFMM/PEG/FSMN-F^-^ groups (n = 3, *P* > 0.05, two-way ANOVA with Šídák’s multiple comparisons test).

The biocompatibility of the fully crosslinked HFMM/PEG/FSMN-F^-^ hydrogel was confirmed through both leachate and direct contact studies (Figure 8). HGECs cultured with the hydrogel leachate for 48 h showed proliferation rates comparable to the control group, suggesting that the thiol ene photocrosslinking process did not produce toxic byproducts.

### 3.5 *In vitro* anti-*Streptococcus mutans* efficacy

#### 3.5.1 Biofilm inhibition confocal and CFU

**FIGURE 9:**
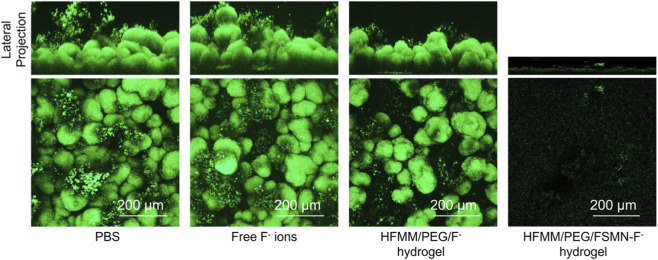
Confocal images of *Streptococcus mutans* biofilm inhibition capacity.

The spatial distribution and structural integrity of the cariogenic biofilms were evaluated across four distinct treatment groups (Figure 9). As shown in the PBS and free fluoride ion groups, *S. mutans* formed highly dense and confluent biofilms characterized by mushroom-like structures. The lateral projections in these control groups confirmed a significant vertical thickness, indicating that neither the absence of treatment nor the application of non-sequestered fluoride ions could prevent the maturation of a robust bacterial matrix.

The HFMM/PEG/F^−^ hydrogel group, which contained free fluoride within the polymer scaffold, showed only a marginal reduction in biofilm accumulation. While the density of the clusters appeared slightly less confluent than the PBS control, large microcolonies were still clearly visible in both the top-down and lateral views.

In striking contrast, the HFMM/PEG/FSMN-F^-^ hydrogel group demonstrated a near complete suppression of biofilm development. The lateral projection revealed an extremely thin layer of scattered cells with no observable vertical architecture. Similarly, the top-down view showed only a few isolated bacterial cells instead of the dense, multi layered clusters seen in the other groups.

**FIGURE 10:**
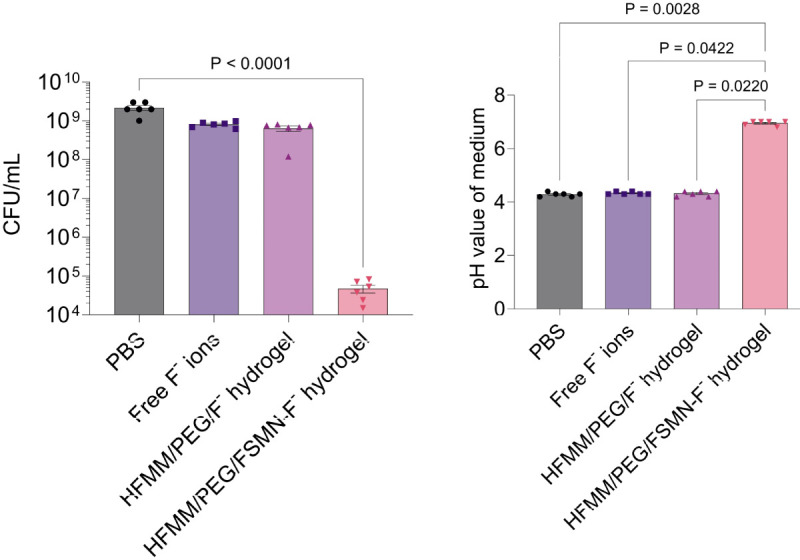
CFU counting of *Streptococcus mutans* biofilm inhibition capacity (left) and culture medium pH value (right) after 48 h (n = 6, one way ANOVA with Tukey’s *post hoc* comparisons).

The results of the CFU assay (Figure 10, left) demonstrated a dramatic reduction in bacterial viability for the HFMM/PEG/FSMN-F^-^ hydrogel group compared to all other experimental conditions. The control group treated with PBS exhibited a robust bacterial load of approximately 2 × 10^9^ CFU/mL, representing the baseline for mature *S. mutans* biofilms on the hydroxyapatite surface.

Interestingly, both the free fluoride ion group and the HFMM/PEG/F^−^ hydrogel group showed only a minimal inhibitory effect, with bacterial counts remaining near 1 × 10^9^ CFU/mL. This lack of significant reduction suggested that neither free fluoride nor fluoride simply doped into the hydrogel matrix can maintain sufficient local concentration to disrupt a developing biofilm. The rapid diffusion of ions into the surrounding medium likely prevented the achievement of a sustained inhibitory did at the material interface.

In sharp contrast, the HFMM/PEG/FSMN-F^-^ hydrogel achieved a reduction of approximately four to five orders of magnitude, with the bacterial load dropping to roughly 5 × 10^4^ CFU/mL. This massive log reduction was statistically significant with a P value of less than 0.0001. This quantitative shift from 10^9^ CFU/mL to 5 × 10^4^ CFU/mL represents a near total elimination of the cariogenic population within the biofilm.

#### 3.5.2 Biofilm acidogenesis

As shown in Figure 10 (right), the pH of the culture medium was measured after the biofilm growth period to evaluate the functional impact of the sustained fluoride release on bacterial metabolism.

In the PBS control group, the pH dropped to approximately 4.2, reflecting a highly acidic environment conducive to enamel erosion. Similar results were observed for the free fluoride ion group and the HFMM/PEG/F^−^ hydrogel group, both of which exhibited pH values around 4.3. These findings confirmed that without a sustained and localized delivery mechanism, the fluoride concentration remained insufficient to inhibit the glycolytic activity and subsequent acid production of the mature *S. mutans* biofilm.

In contrast, the HFMM/PEG/FSMN F hydrogel group successfully maintained the medium pH at a near neutral level of approximately 7.0. The ability of the composite hydrogel to prevent acid accumulation directly correlated with the massive log reduction in bacterial viability and the total disruption of biofilm architecture observed in the previous assays.

## 4 Discussion

### 4.1 Clinical significance of fluoride in oral health

Fluoride ions played a crucial protective role in the maintenance of dental integrity (El-Adl et al., 2025). Achieving the sustained release of fluoride ions within the oral cavity was essential for inhibiting tooth demineralization and promoting the remineralization of existing lesions. This was particularly relevant in orthodontic applications, where the presence of brackets and wires led to the formation of white spot lesions due to plaque accumulation (Liu et al., 2025). By maintaining a stable local concentration of fluoride, the HFMM/PEG/FSMN-F^-^ system can effectively prevent these complications and support the repair of enamel defects (Liu et al., 2025).

### 4.2 Limitations of traditional adsorbents and the role of hydrogels

Various materials have been explored for fluoride ion adsorption and release, including traditional metal hydroxides such as activated aluminum hydroxide and salt fluoride foams. However, these conventional materials were often sensitive to local pH fluctuations, prone to degradation or precipitation, and generally exhibit low biocompatibility (Mishra et al., 2024; Sharma et al., 2026). In contrast, hydrogel materials were stable three-dimensional network polymers capable of rapid water absorption and retention (Sharma et al., 2026).

The utility of the HFMM/PEG system was primarily driven by its unprecedented gelation kinetics. As established in our previous work, the simultaneous grafting of methacrylic acid and norbornene groups onto the hyaluronic acid backbone created a synergistic environment for thiol-ene click chemistry (Gao et al., 2021). When compared with traditional methacrylated HA (HAMA) or norbornene-functionalized HA (HANA) alone, the HFMM derivative demonstrated significantly reduced crosslinking times (Gao et al., 2021).

The primary sustained release mechanism for drug carriers based on hydrogels involved the gradual swelling of the hydrophilic network in an aqueous environment, allowing the therapeutic agent to diffuse through the polymer gaps. Our results in Figure 5 suggest that simply doping fluoride ions into the HFMM/PEG hydrogel did not yield sufficient sustained release performance. This limitation likely stems from the fact that fluoride ions did not form strong ionic bonds with the HFMM/PEG polymer matrix. Therefore, we utilized the rapidly photocrosslinked HFMM/PEG hydrogel as a stabilizing carrier for the high affinity FSMN-F^-^ nanoparticles to create a novel composite with superior release characteristics (Mehta et al., 2025).

### 4.3 Structural advantages of mesoporous FSMNs

The structural characteristics of the FSMN nanoparticles were evaluated using TEM and SEM in Figure 1. The FSMN nanoparticles served as the functional core of our delivery system. During synthesis, CTAB acted as an organic template to form micelles while the silica shell coats the Fe_3_O_4_ surface, resulting in an irregular mesoporous structure (Li et al., 2018). Mesoporous materials were defined as porous solids with surface pore sizes between 2 and 50 nm. Their exceptionally large specific surface area and controllable pore size distribution significantly improved drug loading capacity and sustained release effects, making them ideal candidates for controlled delivery systems (Li et al., 2018).

The formation of these mesopores was confirmed by the nitrogen adsorption and desorption isotherms, which displayed a type IV hysteresis loop caused by capillary condensation (Svidrytski et al., 2020). The determined average mesopore size of 3.1 nm, combined with a specific surface area of 282.34 m^2^/g, provides ample space for fluoride sequestration. Furthermore, the mesoporous silica shell served a dual purpose by preventing the aggregation of the magnetic Fe_3_O_4_ cores and protecting them from oxidation in the complex oral environment (Muzzio et al., 2021; Wang et al., 2020).

### 4.4 Synergistic reservoir-plus-scaffold release mechanism

Our previous research indicated that free FSMN-F^-^ nanoparticles exhibited robust fluoride adsorption but were subject to mechanical loss in dynamic environments (Li et al., 2021). While these nanoparticles were superparamagnetic and could be recovered using magnets, their localized retention in the oral cavity was naturally low. By immobilizing the FSMN-F^-^ nanoparticles within the ultra-fast curing HFMM/PEG hydrogel, we successfully addressed this retention issue (Li et al., 2021).

This ultra-fast *in situ* gelation property was a critical advantage for medical applications. It allowed the material to be applied via a spray device, where it instantly adhered to the tissue surface before being washed away by physiological fluids. For oral cavity applications, where salivary flow and tongue movement were constant, this rapid stabilization ensured that the fluoride reservoir remained localized at the defect site. Furthermore, the absence of an oxygen-inhibition effect in the thiol-ene reaction ensured that the surface of the sprayable gel cures as thoroughly as the bulk material, providing a uniform protective barrier (Zhang et al., 2026a).

Although the hydrogel matrix provided some physical impedance to diffusion, the cumulative release of hydrogel-only group remained high, reflecting the lack of strong chemical affinity between the ions and the polymer chains (Figure 5). The free FSMN-F^-^ nanoparticles showed a more controlled release profile compared to the HFMM/PEG/F^−^ group. In contrast, the release curve of the HFMM/PEG/FSMN-F^-^ hydrogel was significantly flatter and more stable than that of the simple fluoride doped hydrogel. The composite system released only 30%–34% of fluoride ions within the first 3 h and reached the 60% threshold after 12 h. By 96 h, the cumulative release reached 80%, with the curve eventually approaching 90% equilibrium. Most notably, the HFMM/PEG/FSMN-F^-^ hydrogel maintained an effective therapeutic fluoride concentration for up to 168 h. This capability to provide stable, long-lasting fluoride release for 1 week made this system a promising new agent for the prevention and treatment of enamel demineralization and defects around orthodontic brackets (Islam et al., 2025; Mehta et al., 2025).

### 4.5 Promising anti-*Streptococcus mutans* efficacy

The anti-*S. mutans* efficacy observed in this study was a direct consequence of the localized and sustained release of fluoride ions at the material interface provided by the synergistic reservoir plus scaffold architecture. The initial burst release of fluoride from the hydrogel provided only temporary inhibition, allowing the bacteria to colonize and form thick biofilms once the local fluoride concentration drops below the therapeutic threshold. Quantitative analysis revealed a significant four to five log reduction in bacterial viability within the HFMM/PEG/FSMN-F^-^ group compared to the control groups where free fluoride or simple hydrogel doping failed to provide adequate inhibition due to rapid ion diffusion (Fang et al., 2022; Zhou et al., 2021).

The reduction in bacterial load was further corroborated by the confocal microscopy images which displayed the total disruption of the three-dimensional biofilm architecture and the absence of the dense clusters that were prevalent in the PBS and free ion controls. This dramatic reduction in biofilm formation was a direct result of the sustained release capability of the FSMN-F^-^ nanoparticles immobilized within the HFMM scaffold. By maintaining a constant inhibitory concentration of fluoride at the material interface for over 1 week, this system effectively blocked the initial stages of bacterial attachment and prevented the synthesis of the extracellular polysaccharide matrix required for biofilm maturation. These results highlighted the breakthrough potential of the HFMM/PEG/FSMN-F^-^ system as a long-lasting protective barrier against cariogenic challenges in the oral cavity. The superior performance of the HFMM/PEG/FSMN-F^-^ system was attributed to the synergistic reservoir-plus-scaffold mechanism. By anchoring the fluoride pre-adsorbed FSMNs within the ultra-fast-curing HFMM matrix, the system created a high-density antimicrobial zone at the site of application. The steady, long-term release of fluoride ions prevented the initial adhesion of *S. mutans* and continuously inhibits the metabolic pathways required for biofilm maturation (Zhang et al., 2026b).

Crucially, the maintenance of a neutral pH near 7.0 by the composite hydrogel system effectively neutralized the acidogenic potential of *S. mutans* which otherwise dropped to a highly demineralizing pH of approximately 4.2 in the control environments. Therefore, by suppressing the metabolic activity and population growth of *S. mutans*, the HFMM/PEG/FSMN-F^-^ system effectively eliminated the primary driver of enamel demineralization. The failure of the HFMM/PEG/F^−^ group to achieve similar inhibitory results underscored the necessity of the Ni^2+^ chelated mesoporous carrier which prevented the characteristic burst release and washout of fluoride ions. Consequently, by suppressing both the population density and the metabolic acid production of cariogenic bacteria, the HFMM/PEG/FSMN-F^-^ system demonstrated a superior ability to inhibit biofilm virulence and protect dental enamel from mineral loss in complex oral environments (Qiu et al., 2024; Uemura et al., 2023).

Results in this manuscript were particularly significant for clinical applications such as orthodontic treatment, where preventing a drop in local pH was essential for avoiding white spot lesions. The combination of ultra-fast gelation for localized retention and high-capacity fluoride release provided a robust defense against the acidic challenges posed by cariogenic biofilms (Xia et al., 2025).

### 4.6 Limitations and future directions

We acknowledge that while the saliva coated HA disk model provides a controlled environment, it does not fully replicate the extreme physiological fluctuations in the animal model/human oral cavity, such as dietary changes and varying salivary flow rates. Additionally, this study focused on a single species biofilm of *S. mutans*.

Upcoming research will focus on the evaluation of this sprayable hydrogel system in multi species biofilm models, including cross kingdom interactions between fungi and bacteria. Furthermore, we have explicitly mentioned our plan to transition into *in vivo* rodent caries models to evaluate the long-term clinical stability and efficacy of the HFMM/PEG/FSMN-F^-^ system in a living environment.

## 5 Conclusion

In this study, we successfully engineered a sophisticated hybrid delivery system that effectively bridges the persistent gap between high-capacity therapeutic ion loading and clinical retention within dynamic biological environments. The HFMM/PEG/FSMN-F^-^ system achieved a superior sustained fluoride release and a significant four to five log reduction in *Streptococcus mutans* viability while successfully maintaining a neutral pH environment near 7.0 to shield dental enamel from demineralization. The clinical significance of this high-performance biomaterial stems from its ultra-fast gelation kinetics, which enable sprayable application, ensuring immediate stabilization and resisting clearance within the dynamic oral environment. By combining robust ion sequestration with immediate *in situ* crosslinking, this platform provides a transformative strategy for the long term prevention of white spot lesions and the management of complex oral biofilms.

## Data Availability

The raw data supporting the conclusions of this article will be made available by the authors, without undue reservation.
